# The perspectives of people with lived experience, families, and researchers with the manuscript writing process in mental health and substance use research: a qualitative study

**DOI:** 10.1186/s40900-025-00802-3

**Published:** 2025-11-13

**Authors:** Natasha Y. Sheikhan, Kerry Kuluski, Melissa Hiebert, Charlotte Munro, Vivien Cappe, Mary Rose van Kesteren, Sean Kidd, Lisa D. Hawke

**Affiliations:** 1 Institute of Health Policy, Management, and Evaluation, University of Toronto, Toronto, ON Canada; 2 Centre for Addiction and Mental Health, Toronto, ON Canada; 3 Trillium Health Partners, Mississauga, ON Canada; 4 Department of Psychiatry, University of Toronto, Toronto, ON Canada

**Keywords:** Patient engagement, Family engagement, Qualitative research, Mental health, Substance use, Patient and public involvement

## Abstract

**Background:**

Engaging people with lived experience and their families (PWLE/F) as partners in mental health and substance use research can have a positive impact. One way to understand this engagement process and its impact is by reporting on it in peer-reviewed papers. However, little is known about the experiences of PWLE/F and researchers regarding the reporting of engagement activities in peer-reviewed papers and navigating the writing process for such publications.

**Methods:**

This qualitative descriptive study aimed to explore the experiences of PWLE/F and researchers with the writing process for engagement in mental health and substance use research. Separate from participants, PWLE/F were engaged in all phases of the study as partners during the research process. Interviews were conducted with 13 PWLE/F and 12 researchers across Canada via Zoom. Interviews were analyzed using reflexive thematic analysis.

**Results:**

Three themes captured the experiences of PWLE/F: navigating uncharted territories, the disclosure dilemma, and paving the way for meaningful co-authorship. Four themes represented the experiences of researchers: prioritizing informed co-authorship, frustrations with reporting on engagement, navigating how and when to report on engagement, and finding clarity amidst ambiguity.

**Conclusions:**

This study sheds light on the complex experiences of PWLE/F and researchers with the writing process for engagement-focused papers. Overall findings indicate a need for clear guidance for both researchers and PWLE/F involved in mental health and substance use research projects, particularly around what to report on and how to meaningfully collaborate in peer-reviewed papers.

**Supplementary Information:**

The online version contains supplementary material available at 10.1186/s40900-025-00802-3.

## Abstract (plain language summary)

Engaging people with lived experience and families as partners in mental health and substance use research can make the research more relevant and better aligned with community needs (this is referred to as ‘lived experience engagement’, or just ‘engagement’). One way to understand engagement better is by describing it clearly in journal articles. Yet, little is known about how people with lived experience/families and researchers actually experience that writing process. We interviewed 25 people across Canada (13 people with lived experience/families and 12 researchers) to better understand their experiences with the writing process in mental health and substance use research. In our interviews, people with lived experience/families often felt unsure about how they could add to academic papers and faced power imbalances in research settings, such as when researchers used jargon that limited participation. They also talked about what we call the ‘disclosure dilemma’, where they debated the benefits of disclosing their lived experience on papers with the risks of disclosing it due to stigma. On the other hand, researchers valued informed co-authorship with people with lived experience/families, but found it hard to describe engagement in their papers within strict word limits and little tailored guidance to their field. Overall, our study sheds light on a need for clear guidance around the writing and co-authorship process that is specific to mental health and substance use research.

**Supplementary Information:**

The online version contains supplementary material available at 10.1186/s40900-025-00802-3.

## Background

There is a growing movement towards engaging people with lived experience and their families (PWLE/F) as partners in mental health and substance use (MHSU) research [1]. This concept is often referred to as PWLE/F engagement, however, the terminology used to describe this work varies across contexts, with terms such as ‘patient engagement’, ‘patient and public involvement’, ‘co-design’, and ‘service user engagement’ often being used interchangeably. There is considerable variability with how engagement is referred to and defined [2, 3]. For example, in Canada, the Strategy for Patient-Oriented Research defines engagement as active and meaningful partnerships with PWLE/F throughout a research project, from designing a study to dissemination [4]. In the United Kingdom, engagement has been incorporated into Patient and Public Involvement and Engagement (PPIE), with the ‘E’ representing active and meaningful knowledge mobilization and dissemination. This variability has made it difficult to synthesize and appraise research on engagement (e.g., for systematic reviews) [1].

Despite a strong evidence base on engagement in health research, there are key facets of MHSU research that differentiate it from the broader health research field. The distinct context of MHSU research is influenced by the pervasive presence of power imbalances and coercive practices in psychiatry, the perceived vulnerability of PWLE fueled by persistent paternalistic attitudes in psychiatry, and the stigma around the capacity of PWLE to consult in research projects [1, 5, 6, 7]. Moreover, power and privilege continue to undermine engagement in MHSU research, particularly for racialized groups whose knowledge is often marginalized [6]. However, engagement in MHSU research can challenge stigma and shift power dynamics in a positive direction [1].

Recent evidence reviews demonstrate the positive impact that PWLE/F engagement has in MHSU research, such as improving research outputs (e.g., recruitment, dissemination), increasing research quality (e.g., rigour, validity, reflexivity), and improving the research environment (e.g., destigmatization of research) [8, 9, 10]. Engagement can also have a positive impact on individuals, such as the PWLE engaged and the researchers conducting the studies [1, 11, 12]. Although limited, a few studies have also noted the negative impacts of engagement, such as the challenges encountered by PWLE due to limited guidance [13] and the increased resources required for engagement [11]. There remains limited research specific to family engagement in MHSU research [9].

Although there is some evidence around the impact of engagement in MHSU research, there are ongoing debates and variations in the way engagement is written about in peer-reviewed publications and in the way engagement takes place in the reporting process [1]. Variations in reporting hinder our ability to fully understand the current evidence regarding engagement in MHSU research, including engagement experiences and both positive and negative impacts [14]. In addition to variable reporting on engagement in MHSU research publications, practices around including PWLE/F partners as co-authors vary; while some publications explicitly include them as co-authors, others limit recognition to acknowledgements or do not clarify whether authors are PWLE/F. Involving PWLE/F as co-authors on peer-reviewed publications is increasingly recognized as a best practice in research—it is seen as a way of promoting both transparent reporting and fair recognition for the contributions made by PWLE/F to the research [2, 13, 15]. There is, however, a lack of consensus around how and when to include PWLE/F as co-authors. In particular, there is an added layer of sensitivity around MHSU given the intersection with stigma and disclosure [16]. There is some discussion around ethical issues regarding co-authorship with PWLE in health research [2, 10, 17]; however, the literature remains limited concerning the experiences of PWLE/F engaged in MHSU research as co-authors.

It is therefore critical to address the lack of consistent and comprehensive reporting related to engagement in MHSU research, including gaps around the co-authorship process. However, it is first important to understand the perspectives of engagement experts, including researchers and PWLE/F partners, when writing about engagement and when engaging in the co-authorship process in MHSU research. Therefore, the present study seeks to answer the following research question: What are the experiences of PWLE/F engaged and researchers during the manuscript writing process in MHSU research?

## Methods

### Design

This study is part of a three-paper doctoral research project based out of the University of Toronto in Toronto, Canada. A qualitative descriptive approach was used to understand the experiences of PWLE/F partners and researchers with the lived experience-engaged writing process. Qualitative description was selected as it remains close to the participants’ accounts and produces findings that are accessible to practice, while involving low-inference interpretations [18]. Pragmatism—an approach commonly associated with qualitative description, and more recently, patient engagement—underpins this study [19]. Pragmatism is an action-oriented framework that emphasizes practicality and democratic values, using the best methods to research real-world problems while seeking outcomes that are useful to communities [19]. Pragmatism was chosen as it is oriented towards real-world practice, with an epistemological focus on practicality and “what works” [19]. Pragmatism informed our design choices, sampling strategy, and guided our use of reflexive thematic analysis in our analytic plan.

### Setting

The Canada-wide study took place online via the Zoom teleconferencing system. A virtual setting was chosen to increase the coverage of geographically diverse participants across Canada. Although the study is situated online, the research team is based at the University of Toronto, a major international university situated in an urban setting.

### Research team and engagement

Interviews were conducted by the first author (NYS), a doctoral candidate who identifies as a PWLE and has a Master’s degree in Public Health. NYS had previous experience with both qualitative and patient-oriented research. As the first author had lived experience related to the subject matter (inevitably shaping data collection and analysis), this was balanced by ongoing feedback from the doctoral committee and the lived experience advisory group. Given the small networks in engagement and MHSU research, NYS had pre-existing professional relationships with 2 out of the 25 participants in the study. All participants were made aware of the study goals and the nature of the doctoral research study. A description of engagement for this study is shown in Table 1 using the Guidance for Reporting Involvement of Patients and the Public (GRIPP2) short form checklist.

**Table 1:** Guidance for reporting involvement of patients and the public (GRIPP2) reporting checklist

Section and topic	Item
1: Aim	The aim of engagement for this project was to inform the design, analysis, interpretation, and write-up of a study on the experiences of PWLE/F partners and researchers with the writing process.
2: Methods	PWLE/F were involved in all phases of the study. This included a PWLE partner on the first author’s doctoral committee and a PWLE/F Advisory Group that was composed of 2 PWLE and 1 family partner, in addition to the first author identifying as a PWLE. PWLE/F partners were recruited through the Centre for Addiction and Mental Health. Advisory group members were compensated through funding from the Ontario SPOR Support Unit and CIHR.
3: Study results	PWLE/F partners had several positive impacts on the study. PWLE/F ensured the interview guide was relevant, improved recruitment, informed and improved data analysis and the discussion section, and promoted reflexivity. Members of the PWLE/F Advisory Group reported that engagement was a collaborative process in which they felt valued and appreciated, were able to learn from each other, were treated like equals at the table, and found the advisory group dismantled power differences. Members of the advisory group also appreciated how the research lead showed them the data and took the time to work through the analysis together, as it gave them a richer understanding of the study.
4: Discussion and conclusions	Overall, engagement was seen as a positive and impactful experience for all team members. Efforts were made to balance group dynamics such that everyone had an equal voice during meetings and offline comments. A major barrier was limited funding and resources (e.g., for training) given that it was a doctoral dissertation.
5: Reflections/critical perspective	Facilitators for engagement included open communication, flexibility around scheduling and ways to contribute, frequent check-ins, shared power and decision-making, strong relationship-building, institutional buy-in, and support/advocacy from researchers. Advisory group members did not experience any barriers, however, there were institutional barriers to involving a PWLE as a formal member of the doctoral committee — this included initial resistance from the university department to formally recognize a PWLE as a committee member as they were not a faculty member. We addressed this barrier by challenging the status quo in order to ensure the PWLE partner was treated as an equal member of the doctoral committee with voting rights.

### Participants

This study included PWLE partners, family partners, and researchers across Canada who have experience with engagement in MHSU research. Eligible PWLE/F participants had to have been engaged as a PWLE/F on a research team in MHSU research within the last three years and have contributed to an academic journal article related to MHSU research as a co-author or in an acknowledgement. Eligible researcher participants had to primarily conduct research in the MHSU field, have experience engaging PWLE/F within the last three years, and have experience with writing about engagement in academic journal articles.

### Data generation

Data were collected through semi-structured, one-on-one interviews with PWLE/F partners and researchers. Guided by pragmatism, semi-structured interviews were used to gain an in-depth understanding of participants’ knowledge, attitudes, and beliefs regarding the writing experience. Interviews ranged from 25 to 80 min, averaging 51.4 min. Recruitment and interviews occurred from April 2024 until August 2024. Purposive and snowball sampling techniques were used to obtain a diverse sample to develop a holistic understanding of the phenomenon [20]. We adopted a purposive sampling strategy to capture a diverse selection of perspectives across a variety of characteristics, including expertise/role (PWLE, family, researcher), gender, ethnicity, and geographic location. Key informants were identified for snowball sampling purposes to recruit participants within their networks. PWLE/F partners contributed to recruitment by reviewing recruitment materials and disseminating it to their networks, which ultimately broadened our reach and improved inclusivity.

Participants were recruited via email and social media through: (a) a community call-out to research teams across Canada with engagement experience via email and social media; (b) contacting key informants (e.g., engagement leads at organizations, authors of relevant MHSU articles, and from personal networks) to disseminate information regarding the study; and (c) contacting participants in a previous study on best practices guidelines for engagement in MHSU research who consented to be contacted about future research. We aimed to recruit 15 to 25 participants. The sample size was determined based on information redundancy (a concept used to describe when no new information is produced from the data), feasibility, and the availability of eligible participants; this reflects sampling as a pragmatic practice rather than one guided by saturation [21].

Research Ethics Approval was received from the University of Toronto. Informed consent was obtained for all participants. Participants completed a demographic form via REDCap before attending the interviews. Participants received a $50 gift card for participating in the interviews. Interviews were audio-recorded using Zoom with permission from the participants, transcribed using Zoom transcription and edited by the first author, de-identified, and uploaded into NVivo 14 by the first author for analysis. Lastly, memoing and annotations were conducted by the first author during transcription and analysis to enhance reflexivity and trustworthiness [22, 23]. Memoing and annotations were a means of documenting how the researcher’s lived experience shaped interpretation, for example, one memo noted how the primary researcher was sensitive to participants’ discussions of disclosure and stigma, which led to discussions with their co-supervisors to ensure that interpretations were grounded in the data.

### Interview guide

A semi-structured interview guide was designed using the Patient Engagement in Research (PEIR) framework by Hamilton et al. [24], a scoping review by Sheikhan et al. [18], feedback from the Lived Experience Advisory Group, and feedback from NYS’s doctoral committee. The interview guide was modified for two groups due to feedback from PWLE/F partners: PWLE/F partners and researchers. This was because PWLE/F and researchers can have different roles in the manuscript process, which required a few distinct (yet parallel) questions to fully capture their experiences. Interview guide questions aimed to identify and describe participant views regarding their experience with writing about engagement and co-authorship. The interview guide was pilot-tested with 3 PWLE/F and 2 researchers within the network of the investigator to determine the appropriateness and relevance of the questions, and to make any necessary revisions to the guide before the study. For example, pilot-testing with the PWLE/F partners led to adding questions on the authorship process and modifying the language to be more lay-friendly. The interview guides can be found in Appendix A.

### Data analysis

Interview transcripts were analyzed separately using reflexive thematic analysis, following Braun and Clarke’s six-phase approach [25]. Although commonly associated with constructivist and essentialist worldviews, thematic analysis also aligns with pragmatism as it is flexible and encourages making a practical choice regarding which analytical route to take [26]. An inductive approach to thematic analysis was taken. This is a pragmatic choice as it is a data-driven approach, where themes, defined as patterns of meaning, are derived from the data content [25, 26]. Coding was conducted by the first author to elicit descriptive codes that are close to the data content, primarily at the semantic level. While the analytic process was inductive and data-driven, we acknowledge that the primary researcher’s lived experience played a role in interpreting the data. Consistent with Sandelowski [18], we recognize that all qualitative descriptions entail interpretation, even while staying close to the data content.

Reflexive thematic analysis occurred in six iterative phases. Phase 1 involved gaining familiarity with the data, Phase 2 involved developing initial codes from the data, Phase 3 involved searching for themes and subthemes, Phase 4 consisted of reviewing the themes in relation to the data, Phase 5 entailed defining themes and extracting quotes, and Phase 6 involved producing the report [25]. Phases 3 to 6 included ongoing feedback from members of the first author’s doctoral committee and the PWLE/F Advisory Group; here, the first author presented the themes and subthemes they generated from the data to each group through mind-mapping and by sharing the coding hierarchy on NVivo for discussion. Moreover, throughout the process, the first author made reflective notes on power, authorship practices, and disclosure and discussed this with the research team.

## Results

### Participants

Participant characteristics—including gender, roles, ethnicity, and education—are shown in Table 2. Of the 25 participants interviewed, 13 identified as PWLE/F and 12 as researchers. The median age was 39.33 for PWLE/F and 39.75 for researchers. The sample was split evenly between participants in the Canadian province of Ontario where the study was situated and participants from other Canadian provinces. Participant results are organized in two separate sections: PWLE/F experiences and researcher experiences. Exemplar quotes from all 25 participants were used to support the thematic narrative and selected based on feedback from the PWLE/F partners and PhD supervisors.

**Table 2:** Participant demographics

Demographic	PWLE/Family (*n* = 13)	Researchers (*n* = 12)
**PWLE/Family**		
Only PWLE partner/advisor	9 (69.2%)	N/A
Only Family partner/advisor	1 (7.7%)	N/A
Both	3 (23.1%)	N/A
**Researchers**		
Early-career researcher	N/A	8 (66.7%)
Mid-career researcher	N/A	2 (16.7%)
Clinician-scientist	N/A	2 (16.7%)
**Length of time being engaged in MHSU research or length of time conducting engagement in MHSU research**		
1–5 year	7 (53.8%)	7 (58.3%)
6 + years	6 (46.2%)	5 (41.7%)
**Location**		
Ontario	7 (53.8%)	6 (50.0%)
British Columbia	5 (38.5%)	3 (25.0%)
Alberta	0	2 (16.7%)
Quebec	0	1 (8.3%)
Saskatchewan	1 (7.7%)	0
**Age**		
Range	24–63	26–57
Average	39.33	39.75
**Gender**		
Man	4 (​​30.8%)	4 (33.3%)
Woman	7 (53.8%)	8 (66.7%)
Genderqueer/non-binary	2 (15.4%)	0
**Education**		
Some post secondary	1 (7.7%)	0
Post-secondary degree/diploma (College, University, Postgraduate certificate)	9 (69.2%)	0
Professional/clinical degree	1 (7.7%)	2 (16.7%)
Graduate degree	2 (15.4%)	2 (16.7%)
Doctoral degree	0	9 (75%)
**Ethnicity**		
Racialized	6 (46.2%)	4 (33.3%)
Non-Racialized	7 (53.8%)	8 (66.7%)

### Key PWLE/F experiences

**Fig. 1:**
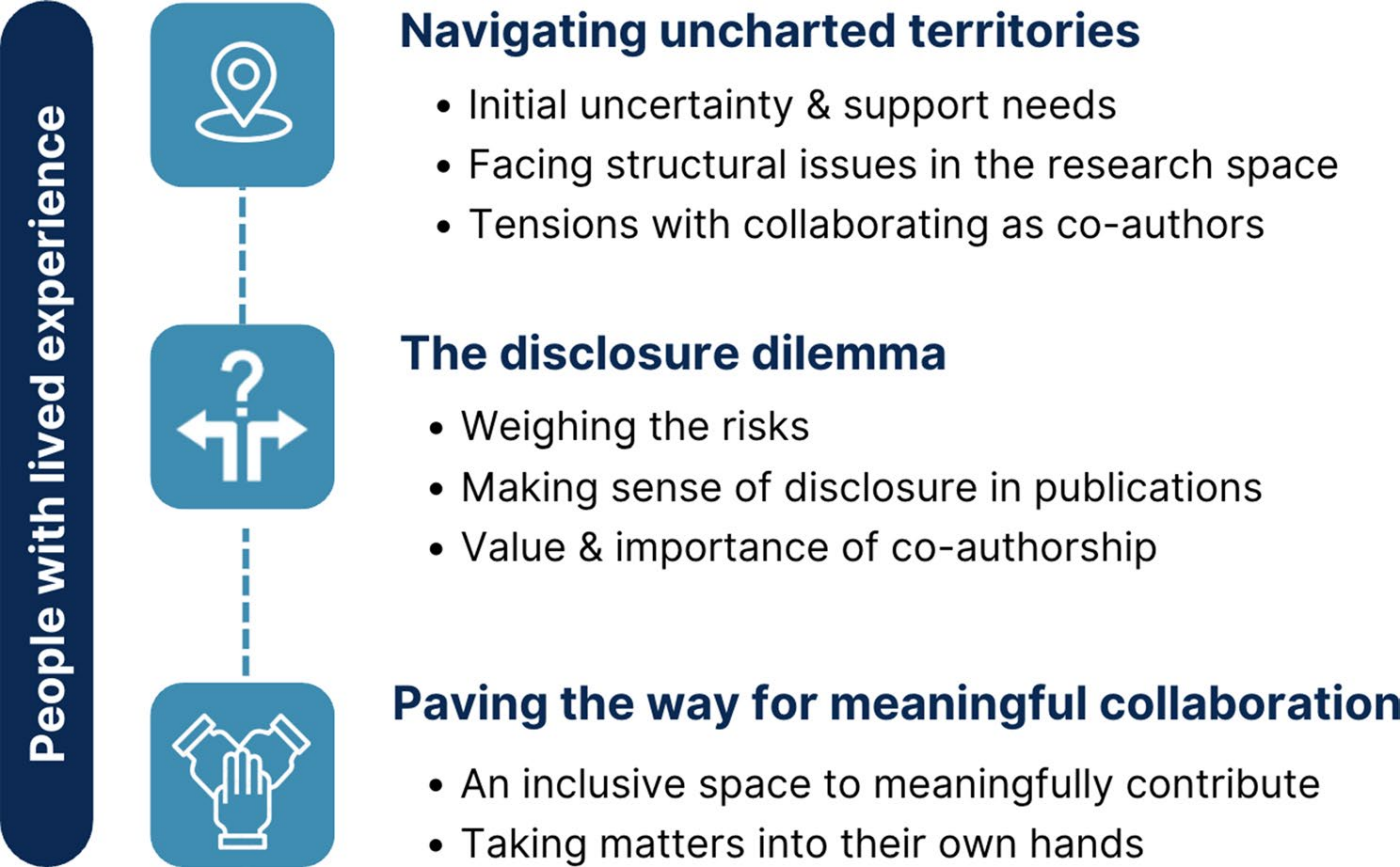
Summary of themes for people with lived experience and family participants

A summary of the themes for PWLE/F is shown in Fig. 1. PWLE/F partners focused on their experiences contributing to MHSU research papers in co-authorship or acknowledgement roles. Three key themes represented their experiences: (1) navigating uncharted territories; (2) the disclosure dilemma; and (3) paving the way for meaningful collaboration.

#### Theme 1: Navigating uncharted territories

This theme encompasses the new and ongoing challenges PWLE/F faced with co-authorship, which included subthemes that unpack the initial uncertainty and support needs they had, structural issues they faced in the research space, and tensions with collaborating as co-authors.

##### Initial uncertainty and support needs

PWLE/F described initially feeling uncertainty, confusion, and self-doubt over how they could contribute to publications early on in the co-authorship process. PWLE/F felt unfamiliar with research as it was a new learning experience for them:

This was fairly new for myself as well. Having to go through that process, because going into it I didn’t think it was one of my strong suits. I kind of associate certain academic things with books. (P13)

PWLE/F felt they needed more time and support early on in their research role as they actively searched for direction and clarity. A few described needing more details in other papers about the engagement process so they could learn from the literature, especially for PWLE/F leading projects for the first time.

##### Facing structural issues in the research space

PWLE/F described larger, systemic issues related to power differences in the research space. Several perceived the academic space to be exclusionary by design and “naturally restrictive” (P24), ranging from the overemphasis on institutional hierarchies to the “scientific jargon” (P04) used in meetings and academic writing. Even what might appear to be a smaller issue for researchers, such as PWLE/F not having an institutional email, deepened power imbalances during co-authorship (e.g., not being taken as seriously when emailing from a non-academic email):

It sucks but people will not respond to my email because I’m not coming from a [*university*] email. (P05)

##### Tensions with collaborating as co-authors

Many PWLE/F were critical of the authorship process, describing issues with having several co-authors and the sequencing of authorship lists. Several PWLE/F felt like they were not meaningfully involved (e.g., brought on as a co-author when the paper was already finished), and that “if you’re bringing people in as a co-author, then they need to actually be given opportunities to co-author it” (P02). For instance:

The fact that we’re constantly brought into research that is like, half or almost done, we can accumulate authorship easily compared to like the traditional academic route. But is it meaningful even to us? I’ve seen a lot of peer researchers or people with lived experience in the research engagement world now questioning a little bit more, like ‘Do I want my name associated with this? Because I might not have agreed with how they engaged me.’ So it seems like the more seasoned the research engagement folks get, the more skeptical and critical they become of the process that engages them. (P09)

Some noted a disconnect between what was written in papers compared to what happened, such as tensions and microaggressions being “invisibilized or erased” in publications (P22). Lastly, a few felt they were put in a box as co-authors, with their other identities and skills (e.g., as a graduate student) ignored:

I myself feel weird being like, ‘Oh, I’m a grad student and I am a person with lived experience.’ Like, I’ll feel weird saying that, and they’ll also wanna just see me as like a person with their experience and that’s all. (P17)

#### Theme 2: The disclosure dilemma

This theme describes the dilemma PWLE/F faced around authorship and disclosure. On the one hand, they had to weigh the risks of authorship due to the complexities of disclosure and stigma. On the other hand, they saw authorship for PWLE/F as valuable not only to themselves, but for advancing lived experience authorship as well.

##### Weighing the risks

Most PWLE/F described feeling hesitant to fully disclose themselves as a PWLE/F co-author on papers due to stigma related to MHSU. Their caution was mainly tied to the permanency of publications (e.g., “being Googlable” (P05) and how the label of PWLE/F might impact their personal and professional life:

Anytime somebody runs a background check on you, and they find [article], you run the risk that whatever you’ve been associated with is going to make that person have a bias towards you. (P20)

When reflecting on their decision not to disclose lived experience in publications, one participant acknowledged that they must contend with the consequences of stigma despite rejecting the responsibility for its creation:

Do I care about stigma? No. Because I don’t create stigma, other people do. That’s not a me problem. That’s on them. But I have to deal with the consequences [of stigma]. So disclosure—so they’re tied in that way. (P22)

PWLE/F had a spectrum of disclosure preferences—some wanted to preserve anonymity as co-authors, while others felt confident being identified as a PWLE/F on papers as they were proud of their lived experience. Disclosure preferences were dynamic and often changed based on where participants were in their personal and professional journeys, such as “applying for grad school” (P17) versus being retired.

##### Making sense of disclosure in publications

Participants viewed disclosure as not only about whether to reveal one’s lived experience in a manuscript, but also how it intersected with authorship practices (e.g., being placed further down the authorship list). PWLE/F made sense of disclosure through discussions around disclosure and labels with researchers. These discussions were seen as important to making an informed decision about whether to disclose, or how much to disclose. A few felt there was a knowledge imbalance between PWLE/F and researchers, with researchers holding more knowledge about authorship and publications than PWLE/F:

I didn’t know what it meant to be in the acknowledgments. Or I didn’t know what it meant to be a co-author on a paper that is indexed somewhere versus not. Or a paper that is, you know, basically paid to publish in some of the lower-quality journals. I didn’t know any of that. And I think researchers most likely know that. (P24)

I don’t think as like collaborators we know what that means…Like what it means to be closer to the front of the authors versus closer to the back, and how can we even utilize that in our careers. (P09)

Here, participants highlighted the complexity of negotiating visibility, with some valuing anonymity, while others felt sidelined when their contributions were acknowledged but not formally recognized.

##### Value and importance of authorship

All PWLE/F felt co-authorship was valuable, providing a positive counterbalance to the risks associated with disclosure:

We’re really putting out there a lot more than we may realize, like kind of that risk. But I think to effect change, someone’s got to step up to the plate. (P04)

Most described the importance of recognizing PWLE/F contributions through co-authorship as PWLE/F “should be getting credit” (P18) for their work. Lastly, several recognized the value of co-authorship for personal goals, as “an accomplishment [they] had not had before” (P20) and for “professional development” (P22).

#### Theme 3: Paving the way for meaningful collaboration.

 This theme highlights the embodiment of best practices by research teams that facilitated meaningful co-authorship, in addition to efforts made by PWLE/F to make the most out of their authorship experience.

##### An inclusive space to meaningfully contribute

An inclusive co-authorship space was rooted in a collaborative writing process and shared values with researchers. This included honest and positive communication from researchers, such as “being very clear about what does or doesn’t belong in a paper, in a respectful way” (P23), and various opportunities to contribute based on needs and skills. PWLE/F also found accommodations and support from researchers helpful. Having ongoing meetings and working sessions, such as going over peer reviews, helped PWLE/F meaningfully contribute to papers:

We were writing together. Like, we actually would meet up in the coffee shop for a few hours and review the transcript she sent me. (P09)

Because it was a team effort, we would sit around and discuss, somebody capturing that data and then putting it together and sharing it. (P13)

Power sharing and being treated as equals further facilitated an inclusive space, such as “not using [professional] titles” (P05) between team members, addressing power imbalances early on, and “being given an equal amount of time to talk and share” (P02).

##### Taking matters into their own hands

In addition to researchers creating an inclusive space, meaningful co-authorship was further crafted by PWLE/F. PWLE/F were proud of their lived experience and leveraged their expertise as they contributed to papers. For some, meaningful co-authorship required doing their own background work:

The process was explained to me, and I did my own research to understand the process. Because without that research, I wouldn’t have been comfortable taking on the title of co-author. (P03)

For others, meaningful co-authorship involved “constantly reflecting on things you could have done better” (P07), remaining critical, and seeing things from different perspectives:

…drawing on academic sources, drawing on my voice, drawing on the voices of the team, drawing on the voices of the people who answered the questions with lived experience… (P13)

Overall, meaningful co-authorship was crafted through the combined efforts of researchers and PWLE/F, highlighting the powers of collaborative partnership.

### Key researcher experiences

**Fig. 2:**
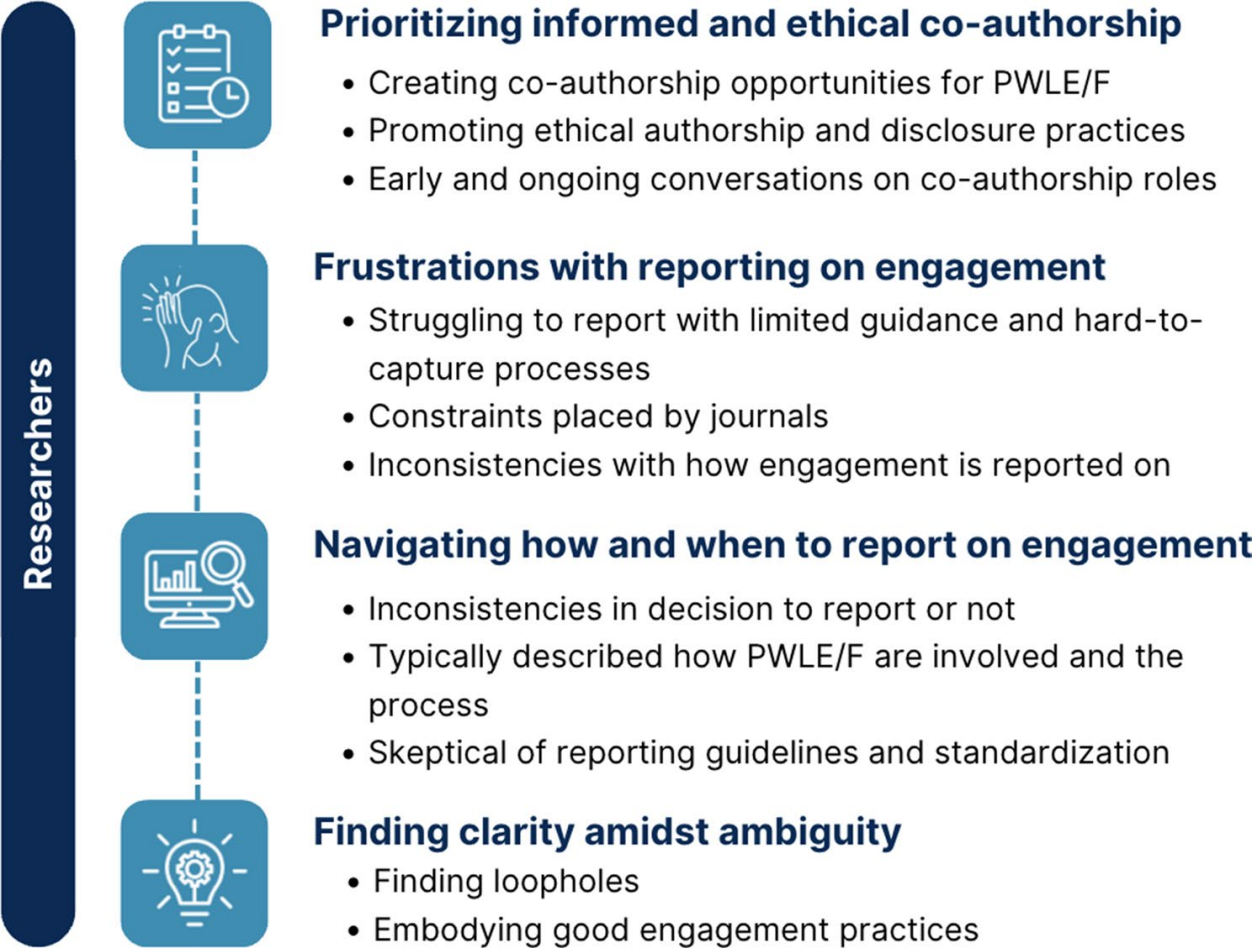
Summary of themes for researcher participants

A summary of the themes for researchers is shown in Fig. 2. As researchers reflected on the manuscript writing process, they addressed engagement experiences, but also emphasized challenges around reporting on engagement. Four key themes represented the experiences of researchers with writing peer-reviewed papers that included an engagement component in MHSU research: (1) prioritizing informed and ethical co-authorship; (2) frustrations with reporting on engagement; (3) navigating how and when to report on engagement; and (4) finding clarity amidst ambiguity.

#### Theme 1: Prioritizing informed and ethical co-authorship

This theme centers around prioritizing informed co-authorship with PWLE/F. Researchers felt it was paramount to have opportunities and discussions around co-authorship that were flexible, ethical, and prioritized PWLE/F needs.

##### Creating co-authorship opportunities for PWLE/F

Researchers emphasized the importance of collaborating with PWLE/F as co-authors on papers and creating opportunities for PWLE/F to meaningfully participate as co-authors. This included properly compensating them for their contribution and not having authorship alone as the sole incentive for engagement (e.g., holding the “publication as just the carrot itself” (P25)). For instance:

Anybody on the team who feels that they meet the ICMJE [International Committee of Medical Journal Editors] criteria, including patient partners, can contribute to a manuscript that we’re writing. (P06)

##### Promoting ethical authorship and disclosure practices

In the context of co-authorship in MHSU research, researchers felt it was critical to prioritize ethical co-authorship and disclosure practices. They described catering to a range of disclosure preferences for PWLE/F partners. This included not ‘outing’ co-authors as PWLE/F without their consent, and ensuring they feel comfortable with what is being disclosed. This was especially relevant for PWLE building their careers:

We’ve had a person with lived experience who did engagement with us who ended up doing a Master’s degree, and now is building a professional career, and would prefer not to be identified in that way. And I think that’s totally reasonable and totally fair. (P16)

Flexibility around co-authorship and labels was central, such as asking PWLE/F “if they wanted to be named by name” (P10) and providing a range of options for how they want to be listed as co-authors:

Giving people choice in how they want to be listed on the manuscript and the authorship team—whether it’s research assistant, partner, or creatively brainstorming with people with lived experience how they would like to be named, and providing flexible options around that if they’re not ready to disclose being a patient partner. (P14)

##### Early and ongoing conversations on co-authorship roles

Lastly, researchers spoke about the importance of having early and ongoing conversations with PWLE/F about their roles and responsibilities as co-authors. This also included discussing with PWLE/F what it means to be a co-author and the permanency of publications, with “the expectations about their contribution needs to be clear from the get-go.” (P11).

#### Theme 2: Frustrations with reporting on engagement

This theme describes the frustrations researchers felt with the reporting landscape, including practical and structural challenges.

##### Struggling to report with limited guidance and hard-to-capture processes

Researchers discussed how they struggled to report on engagement in MHSU research with limited guidance, especially when trying to represent relational, fluid, and context-specific processes. Some reflected on the struggles they experienced with reporting when they were new to engagement research, including feeling uncertainty about what to report on, because “there’s no guide.” (P01). They expressed a need for guidance early on and searched for guidance around what to report on. Several felt there was no clear guidance for reporting and felt “like I was just kind of making it up.” (P19) Some highlighted how there is a need for guidance tailored to engagement in MHSU research to prevent tokenistic reporting:

There’s no clear guidelines of what they want. It’s just like that tick box, right? It’s like, ‘Do you have people with experience on a team or did you do any kind of engagement?’ And it’s kind of easy to check that box. But it’s hard to communicate that work. (P16)

Researchers also felt it was difficult to capture the experiences of PWLE/F; for instance, one researcher commented on how they “shouldn’t be reporting on a patient’s experience based on [their] perception of it.” (P08).

##### Constraints placed by journals

Researchers described feeling frustrated with the constraints placed by journals. This primarily included the word limit placed by journals, with several feeling that “there has to be more space to report on [engagement]” (P16). The limited word count made it harder for researchers to describe engagement activities in any depth:

You’re working within page limits for a research paper, so how can you balance that within it, right? You want to provide as much depth as you can to be transparent about your methodology and your engagement, but at the same time, you have limited space. So I think that’s where I struggle. (P25)

Other barriers related to journals included limited flexibility around co-author names, making it difficult to maintain anonymity for PWLE/F who may not be comfortable with disclosure.

##### Inconsistencies with how engagement is reported on

Lastly, researchers felt frustrated with how engagement is inconsistently reported on in the literature, especially given the lack of standardization within the field. Several described reading papers that gave little to no description of the engagement process, resulting in “a lack of information in a lot of articles about what engagement or partnership actually looked like” (P14). They were also frustrated with the misalignment between the terms used (e.g., “patient engagement”, “co-design”) versus the actual practices carried out:

I found that a lot of studies called something patient engagement, but they’re not actually like engaging the patients. It’s more just like, there are participants basically. (P15)

People report in so many different ways. People say they use co-design, and I’m like, ‘Did you though?’ When I’m reading, I don’t know if this is actually co-designed. (P10)

#### Theme 3: Navigating how and when to report on engagement

This theme highlights the journey researchers undergo when determining how and when to report on engagement in papers, such as factors that influence their decisions and their skepticism towards standardization.

##### Inconsistencies in decision to report or not

Researchers described inconsistent patterns with deciding how and when to report on engagement. Many felt their decision was dependent on a multitude of factors, including the type of journal, the type of paper (e.g., process paper versus original research, qualitative versus quantitative), and the level of engagement on the project:

It really depends. If I’m writing a methods paper, I might put less information and just refer to another paper for more information. I think it depends on the purpose of the research and the amount of information I’m putting in the paper. (P10)

A few mentioned providing minimal details and reflection when reporting on engagement, with one noting they “don’t think [they] put much apart from the fact that they were involved” without any “reflection around it” (P12). Another remarked:

…it’s also not like a [personal] journal, you know, where it’s like, ‘Oh, today I woke up and felt sad.’ It’s not super detailed. It’s just more like here’s how we did it. Here’s how it was done. (P01)

As they navigated how to write about engagement, several researchers noted how important it was “to be clear and transparent” (P25) for honest and ethical reporting.

##### Typically described how PWLE/F are involved and the process

As researchers reflected on how they reported on engagement, most described providing a general overview of the engagement process, often in the methods section, with a description of the PWLE/F engaged:

One of the things that I consistently report on is who the youth or participants were that I engaged in informing the study. (P10)

I just write what we’ve done and try to include as much as I can. (P25)

Some researchers went into more detail, providing a description of “process elements” (P14), the number of meetings held, description of the roles and tasks, and if compensation was provided. A few also reported on the impact or value that engagement had on the research.

##### Skeptical of reporting guidelines and standardization

Some researchers were wfary of reporting guidelines and standardization, feeling that following guidelines may not necessarily mean better engagement:

Just because you followed a guideline, doesn’t mean you did it well. […] I feel like if you have standardized guidelines, you’re trying to fix something that can’t be fixed. It’s like trying to turn all art into paint by number. (P01)

You’re putting a rigid structure on something, and if your engagement doesn’t fit that structure, it can make it look like it was done poorly. When in fact it was probably super meaningful, but didn’t fit that recipe card. (P08)

#### Theme 4: Finding clarity amidst ambiguity

This theme provides an overview of what helped researchers navigate reporting. Researchers proactively developed their own methods to report on engagement, in light of barriers and a lack of tailored guidance.

##### Finding loopholes

To address their frustrations with the reporting landscape, especially with limited word counts, researchers found loopholes to reporting on engagement. Some loopholes included submitting to journals with higher word counts or engagement-focused journals:

I just chose a journal that embraces engagement. They have the requirement that you have to report on it at least—there’s a minimum reporting requirement. And they also don’t have a word limit in terms of fully reporting on engagement. (P08)

When word count was limited, researchers had to “be a bit creative” (P14) and find loopholes such as putting their engagement plan in the appendix or using figures and tables to describe engagement. A few researchers also mentioned citing a process paper or “anchoring paper” (P21) within the methods section that provides more details on the engagement process.

##### Embodying good engagement practices

Most researchers felt that embodying good engagement practices helped them feel more confident with reporting. This included using frameworks and tools to help write about engagement, such as “the PPEET” (P10, P15), also known as the Public and Patient Engagement Evaluation Tool [27]. A few researchers noted continuously reflecting on aspects to report on and had detailed documentation during the engagement process (e.g., through notes):

Luckily, I took really detailed notes about the process of partnership, recorded all our meetings, reflections, and memos along the way, but for someone who’s new, those could be really useful when reporting in the papers. (P14)

Researchers further described embodying engagement practices that helped them collaborate with the PWLE/F co-authors, such as: putting “relationships first” (P21), having a strong infrastructure for engagement, providing training and mentorship for PWLE/F, and scheduling ongoing meetings and check-ins with PWLE/F co-authors. Several researchers felt it was critical to prioritize the preferences of PWLE/F when reporting on engagement:

I don’t think it’s for me to decide. I think it’s for the person to decide. I honestly don’t care what we write in the paper as long as the people are comfortable with it. (P11)

Lastly, a few researchers described learning and “looking at other papers” (P10) to see what to report on regarding engagement in MHSU research.

## Discussion

### Overview

To our knowledge, this is the first qualitative study that explores the experiences of PWLE/F partners and researchers with the MHSU manuscript writing process. The learnings from our study are twofold. First, we unpack the experiences of PWLE/F with co-authorship. PWLE/F have to navigate through uncertainty, tension, and power dynamics as co-authors and in acknowledgments, and face a dilemma with disclosing their lived experience. Second, we explore the experiences of researchers writing with PWLE/F, on PWLE/F-engaged peer-reviewed papers. Researchers described how they prioritized informed co-authorship of PWLE/F, but also how they navigated the challenges of reporting on engagement—including their frustrations with reporting. By elevating the voices of both PWLE/F and researchers, we gain a dual lens on the phenomenon of writing manuscripts in PWLE/F engagement settings.

### Differences in participant responses

PWLE/F in our study mostly focused on their experiences with co-authorship and had limited experiences with reporting on engagement compared to researchers. In contrast, researchers focused more on the challenges of reporting on the engagement process rather than the co-authorship experience. This is likely because most PWLE/F did not have a large role in leading the writing process or drafting the manuscripts, with many not being engaged from the start. In a rapid review that explored patient co-authorship in health research reviews, Ellis et al. [2] found that only 32% of the patient co-authors reported being involved in the writing and publishing phase of the review. PWLE/F may have limited involvement in publications due to the additional time, costs, and resources needed (e.g., training) for co-authorship [28]. Our findings therefore provide some clarity on the limited experiences of PWLE/F as co-authors, reflecting the limited literature on this phenomenon. However, it is important to note that PWLE/F partners who lead papers may have different experiences as they have a different position in the academic hierarchy—additional research is required to further explore the diverse roles PWLE/F have on research teams and how these roles influence their experiences with preparing manuscripts for publication.

### Risk of disclosure or opportunity to destigmatize?

Our findings show that both PWLE/F and researchers felt it was important to create opportunities for co-authorship. These findings reflect a growing shift towards recognizing PWLE/F as co-authors in health research [29, 30], with calls for studies to standardize the use of metatag and keywords to fully understand the extent of patient authorship [17, 28]. However, there are power and knowledge considerations in MHSU research that distinguish it from other areas of health research [31]. In particular, PWLE co-researchers’ choice to disclose their lived experience is heavily influenced by stigma, and it is also subject to change depending on context and life circumstances [32]. Issues with disclosure have also been discussed in the context of researchers with lived experience, with researchers being hesitant to disclose their lived experience due to stigma [16, 33]. Engagement often involves emotional labour and ongoing negotiations around disclosure, with stigma influencing when and how individuals share their lived experience—this showcases the need for structures that safeguard wellbeing [34]. In our findings, PWLE/F and researchers shared a mutual acknowledgement of the sensitivity with disclosing one’s lived experience related to MHSU, suggesting a need for tailored guidance that includes a discussion of disclosure and its implications.

It is important to note that while we highlight the risks of disclosure related to MHSU, we should not let these concerns overshadow the movement towards co-authorship with PWLE/F or overlook how disclosure can contribute to the destigmatization of MHSU challenges. We are simply raising awareness that PWLE/F should have the autonomy to make their own informed decision around disclosure, and that research teams should provide options around co-authorship and acknowledgements that are tailored to the needs of PWLE/F.

### Frustrations with reporting

The frustrations felt by researchers around reporting on engagement, coupled with PWLE/F wanting more information from publications around engagement processes, may suggest the need for guidance around reporting on engagement and co-authorship with PWLE/F that is tailored to MHSU research. While there are evidence-based reporting guidelines such as the Guidance for Reporting Involvement of Patients and the Public (GRIPP) and GRIPP2 checklists that can be used to improve the reporting of engagement in health research [35], there is limited uptake in MHSU research [1]. Moreover, the GRIPP has been critiqued as being limited in terms of reflexive reporting (e.g., reflections on power relations are not promoted), with uncertainty around *who* is completing the checklist (e.g., is it completed with PWLE) [36]. Researchers have also called for expanding the GRIPP2 to record whether there are PWLE co-authors [37]. However, as previously noted, there are risks to identifying PWLE/F co-authors in MHSU research and including it in any reporting guidelines should be approached with sensitivity.

It is noteworthy that some researchers in our study were critical of standardization and reporting guidelines for engagement, mirroring similar critiques of reporting guidelines in qualitative research and how they create a false sense of universality and quality [38, 39]. Standardized reporting might not necessarily equate to higher quality research [39], just as it might not equate to a higher quality of engagement. Participants in our study also emphasized that engagement contexts are unique and fluid, which further challenges the notions of universality. While there is value in reporting guidelines [40], there must be a middle ground that balances guidance and transparency without imposing rigid checklists that overlook the complexities of engagement.

### Facilitators to meaningful co-authorship

Our findings provide solutions that helped PWLE/F and researchers with co-authorship. This reflects best practices for engagement in MHSU research, such as using accessible language, providing training and mentorship for PWLE/F, and power sharing between PWLE/F and researchers [11, 13, 41, 42]. It is important to note that meaningful power-sharing may require structural changes beyond individual projects—universities, funders, and publishers play a major role in creating conditions that value PWLE/F contributions and amplify the lived experience voice. For example, this can be done through recognition of co-authorship, supportive authorship policies, and funding mechanisms that fairly compensate engagement activities such that authorship is not tokenistic. The knowledge gained can be used to inform guidance around co-authorship with engagement to facilitate a meaningful and collaborative space.

### Strengths and limitations

A major strength of this study is PWLE/F engagement. PWLE/F were engaged at various levels, informing study methodology, data collection, data analysis, and producing the manuscript. This study also included a diverse sample of PWLE, families, and researchers across Canada. However, despite having a relatively strong diversity, there is not enough diversity in the field as engagement in research is often critiqued for having limited representation of socially marginalized groups [43]. Moreover, although the virtual setting was beneficial for this study as it increased flexibility and coverage across Canada, it may have limited the participation of individuals who have limited access to the internet or a computer [44]. Moreover, the sample was limited to English-speaking PWLE/F and researchers in Canada, limiting the transferability of the findings to populations inside and outside of Canada that do not fit this demographic profile.

## Conclusion

This study contributes to our understanding of PWLE/F and researchers’ experiences with the manuscript writing process for engagement in MHSU research. We highlight the complexities behind reporting, including the frustrations with reporting for researchers and challenges with the co-authorship process for PWLE/F. Our findings highlight the need for institutions to adopt policies that recognize and support PWLE/F contributions; this includes fair compensation, informed co-authorship practices, and clearer reporting standards. More guidance is needed for both researchers and PWLE/F engaged in MHSU research projects around how to meaningfully collaborate in academic publications and what to report on. Building on this work, we have therefore developed a reporting guidance document for reporting on engagement and co-authorship with PWLE/F in MHSU research [45].

## Supplementary Information

Below is the link to the electronic supplementary material.

Supplementary Material 1

## Data Availability

The full dataset is not available for public review and use due to privacy and confidentiality reasons. Parts of the data generated and analyzed during this study are included in this manuscript as quotations. Please contact the corresponding author for any request regarding the study data.

## Abbreviations

PWLE/F: People with lived experience and families
MHSU: Mental health and substance use research
GRIPP: Guidance for Reporting Involvement of Patients and the Public
