# Supplementary material for: The perspectives of people with lived experience, families, and researchers with the manuscript writing process in mental health and substance use research: a qualitative study
Source: Res Involv Engagem. 2025 Nov 13;11:132. doi: 10.1186/s40900-025-00802-3 (PMC12613716; doi:10.1186/s40900-025-00802-3)
Supplement: Supplementary file 1 — Supplementary Material 1 [file 40900_2025_802_MOESM1_ESM.docx]

# **Interview Guide 1 (Researchers)**

Thank you for taking the time to talk to me today. Your expertise will be invaluable to this study. Please note that this interview may take anywhere from 40 to 80 minutes but can be shorter or longer based on your preference and/or your availability. Before we begin, I would like to repeat some reminders regarding your participation in this study:

1. I would like to affirm that participation in the study is voluntary. It is your choice whether you decide to take part in this study or not. If you do decide to participate, you can skip any question or withdraw from the study at any point during the interview with no negative consequences.
2. The interview will be audio-recorded, but your identity will be anonymized in transcripts and the information you provide during this meeting will be kept confidential. The recordings will only be accessible by members of the doctoral research team and only for study-related activities.
3. Do you give your consent to audio-record this conversation?

This study seeks to understand what researchers, people with lived experience (PWLE) partners, and family partners perceive are valuable components to report on regarding engagement in mental health and substance use (MHSU) research. We define engagement as “active and authentic collaboration with PWLE throughout the research process, from planning to dissemination.” We are interested in PWLE as research collaborators, rather than PWLE as research participants.

In today’s interview, we will first discuss your experiences with reporting on engagement activities when preparing peer-review manuscripts, and then we will explore areas that might be important to include in a reporting guideline. The study findings will be then used to develop a reporting guidance document for engagement in MHSU research at a later phase. The reporting document can be used to provide researchers with a comprehensive set of items to follow when preparing peer-reviewed manuscripts for projects that included an engagement component. Although there are existing guidelines for patient and public engagement in research, none of them are specific to MHSU research.

Do you have any questions before we begin the interview?

### **Section A: Background Questions**

1. Tell me about your experience as a researcher conducting patient-oriented research in the field of MHSU. How might engagement be different in MHSU research compared to other areas of health research?
2. What do you feel should be the goals or priorities of a reporting guideline for engagement in MHSU research?
3. Would you use a reporting guideline when writing up your own research?

### **Section B: Experiences with reporting**

The following questions will focus on your experiences with preparing manuscripts for studies that included an engagement component. To clarify, this can range from a one-time advisory meeting to co-production (*e.g.,* having patients as co-researchers).

1. In general, how helpful have you found reporting guidelines when writing a manuscript? (Example, PRISMA for reviews, COREQ for qualitative research, CONSORT for randomized trials)
2. When writing a manuscript on a research project that included an engagement component, in what circumstances would you include a section on engagement?
3. Can you describe your approach to reporting on engagement activities in peer-review manuscripts?

### **Section C: Exploring reporting items**

The following questions will explore potential areas that researchers might report on when preparing a manuscript on a project that engaged PWLE.

1. What do you feel are important aspects to report on in the engagement of PWLE in MHSU research?
2. Do you feel it is important to provide a background on the research team? What might this include?
3. How can the contributions of PWLE best be captured in manuscripts?
4. When preparing manuscripts for peer review, some researchers suggest that we should also be reporting on the impact engagement might have had on the research project. In your experience, how important is it to report on the impact of engagement when preparing manuscripts for peer review?
5. Are there any ethical considerations you think should be taken into account when reporting on engagement in MHSU research?
6. How can power imbalances be addressed in this reporting guideline?

**Conclusion**

1. Now that we’ve discussed various aspects of a reporting guideline, do you think there would be a benefit of developing one? Do you think you would use it? Why or why not?
2. Is there anything else that you would like to add or emphasize as we wrap up?

Thank you for taking the time to share your thoughts and experiences today. What you have shared will be immensely helpful as we develop the reporting guidance document for engagement in MHSU research. Would you like to provide your email address in order to receive a summary of the study findings when they become available?

# **Interview Guide 2 (PWLE Partners and Family Partners)**

Thank you for taking the time to talk to me today. Your expertise will be invaluable to this study. Please note that this interview may take anywhere from 40 to 80 minutes but can be shorter or longer based on your preference and/or availability. Before we begin, I would like to repeat some reminders regarding your participation in this study:

1. I would like to affirm that participation in the study is voluntary. It is your choice whether you decide to take part in this study or not. If you do decide to participate, you can skip any question or withdraw from the study at any point during the interview with no negative consequences.
2. The interview will be audio-recorded, but your identity will be anonymized in transcripts and the information you provide during this meeting will be kept confidential. The recordings will only be accessible by members of the doctoral research team and only for study-related activities.
3. Do you give your consent to audio-record this conversation?

This study seeks to understand what researchers, people with lived experience (PWLE) partners, and family partners perceive are valuable components to report on regarding engagement in mental health and substance use (MHSU) research. We define engagement as “active and authentic collaboration with PWLE throughout the research process, from planning to dissemination.” We are interested in PWLE as research collaborators, rather than PWLE as research participants.

In today’s interview, we will first discuss your experiences with reporting on engagement activities when preparing peer-review manuscripts, and then we will explore areas that might be important to include in a reporting guideline. The study findings will be then used to develop a reporting guidance document for engagement in MHSU research at a later phase. The reporting document can be used to provide researchers with a comprehensive set of items to follow when preparing peer-reviewed manuscripts for projects that include an engagement component. Although there are existing guidelines for patient and public engagement in research, none of them are specific to MHSU research.

Do you have any questions before we begin the interview?

### **Section A: Background Questions**

1. Tell me about your experience as a PWLE on research projects.
2. Do you think it would be helpful for authors to have guidelines when writing about engagement in MHSU research?
3. What do you feel should be the goals or priorities of a reporting guideline for engagement in MHSU research?

### **Section B: Experiences with reporting**

The following questions will focus on your experiences being involved with preparing/reviewing manuscripts for studies that included an engagement component.

1. Can you describe how you have been involved in writing up or reviewing manuscripts for publication?
2. When you are engaged in a research project, have you been included as an author in a peer-reviewed publication?
3. When you've been involved in reporting on engagement in research, how has engagement been described?

### **Section C: Exploring reporting items**

The following questions will explore potential areas that researchers might report on when preparing a manuscript on a project that engaged PWLE.

1. What do you feel are important aspects to report on in the engagement of PWLE in MHSU research?
2. Do you feel it is important to provide a background on the research team? What might this include?
3. When preparing manuscripts for peer review, some researchers suggest that we should also be reporting on the impact engagement might have had on the research project. In your experience, how important is it to report on the impact of engagement when preparing manuscripts for peer review?
4. How can the contributions of PWLE best be reflected in manuscripts, for example, through co-authorships or acknowledgements?
5. Are there any ethical issues you think should be taken into account when reporting on engagement in MHSU research?
6. How can power imbalances be addressed in this reporting guideline?

**Conclusion**

1. Now that we’ve discussed various aspects of a reporting guideline that’s specific to MHSU, do you think there would be a benefit of developing one? Do you think research teams should use it?
2. Is there anything else that you would like to add or emphasize as we wrap up?

Thank you for taking the time to share your thoughts and experiences today. What you have shared will be immensely helpful as we develop the reporting guidance document for engagement in MHSU research. Would you like to provide your email address in order to receive a summary of the study findings when they become available?
